# Motor neuron diseases caused by a novel *VRK1* variant – A genotype/phenotype study

**DOI:** 10.1002/acn3.50912

**Published:** 2019-09-27

**Authors:** Maryam Sedghi, Ali‐Reza Moslemi, Montse Olive, Masoud Etemadifar, Behnaz Ansari, Jafar Nasiri, Leila Emrahi, Hamid‐Reza Mianesaz, Nigel G. Laing, Homa Tajsharghi

**Affiliations:** ^1^ Medical Genetics Laboratory Alzahra University Hospital Isfahan University of Medical Sciences Isfahan Iran; ^2^ Department of Pathology University of Gothenburg Sahlgrenska University Hospital Gothenburg Sweden; ^3^ Institute of Neuropathology Department of Pathology Institut Investigació Biomèdica de Bellvitge (IDIBELL)‐Hospital de Bellvitge Hospitalet de Llobregat 08907 Barcelona Spain; ^4^ Neuromuscular Unit Department of Neurology Institut Investigació Biomèdica de Bellvitge‐(IDIBELL)‐Hospital de Bellvitge Hospitalet de Llobregat 08907 Barcelona Spain; ^5^ Department of Functional Neursurgery Faculty of Medicine Isfahan University of Medical Sciences Isfahan Iran; ^6^ Department of Neurology Faculty of Medicine Isfahan University of Medical Sciences Isfahan Iran; ^7^ Department of Pediatric Neurology Faculty of Medicine Isfahan University of Medical Sciences Isfahan Iran; ^8^ Department of Genetics and Molecular Biology Isfahan University of Medical Sciences Isfahan Iran; ^9^ Centre for Medical Research The University of Western Australia and the Harry Perkins Institute for Medical Research Nedlands Western Australia Australia; ^10^ School of Health Sciences Division Biomedicine and Translational Medicine University of Skovde Skovde Sweden

## Abstract

**Background:**

Motor neuron disorders involving upper and lower neurons are a genetically and clinically heterogenous group of rare neuromuscular disorders with overlap among spinal muscular atrophies (SMAs) and amyotrophic lateral sclerosis (ALS). Classical SMA caused by recessive mutations in *SMN1* is one of the most common genetic causes of mortality in infants. It is characterized by degeneration of anterior horn cells in the spinal cord, leading to progressive muscle weakness and atrophy. Non‐SMN1‐related spinal muscular atrophies are caused by variants in a number of genes, including *VRK1*, encoding the vaccinia‐related kinase 1 (VRK1). *VRK1* variants have been segregated with motor neuron diseases including SMA phenotypes or hereditary complex motor and sensory axonal neuropathy (HMSN), with or without pontocerebellar hypoplasia or microcephaly.

**Results:**

Here, we report an association of a novel homozygous splice variant in *VRK1* (c.1159 + 1G>A) with childhood‐onset SMA or juvenile lower motor disease with brisk tendon reflexes without pontocerebellar hypoplasia and normal intellectual ability in a family with five affected individuals. We show that the *VRK1* splice variant in patients causes decreased splicing efficiency and a mRNA frameshift that escapes the nonsense‐mediated decay machinery and results in a premature termination codon.

**Conclusions:**

Our findings unveil the impact of the variant on the *VRK1* transcript and further support the implication of *VRK1* in the pathogenesis of lower motor neuron diseases.

## Introduction

Hereditary lower motor neuron diseases are a genetically and clinically heterogenous group of rare neuromuscular disorders within the spectrum spinal muscular atrophy/hereditary motor neuropathy. They are characterized by degeneration of anterior horn cells in the spinal cord, leading to progressive muscle weakness and atrophy.^1^ Clinical course varies depending on the type of SMA. SMA is most often caused by recessive mutations in the survival motor neuron gene (*SMN1*) and it is one of the commonest genetic causes of mortality in infants.^2^ There are, however, a significant number of SMA cases with heterogenous non‐*SMN1*‐related causes. Non*‐SMN1‐*related SMAs, including nonproximal SMA, bulbar palsy SMA, spinobulbar muscular atrophy, and variant infantile SMA characterized by SMA features along with additional symptoms and signs or atypical phenotypes.^3^ Pontocerebellar hypoplasia type 1A (SMA‐PCH) (MIM#607596), is a rare infantile disorder combining SMA with severe congenital microcephaly, mental retardation, and cerebellar signs.^4^, ^5^ Variants in *VRK1*, encoding the vaccinia‐related kinase 1 (VRK1) which is widely expressed in human tissues and has a vital role in embryonic cortical neuronal proliferation,^6^ have been reported in patients with SMA‐PCH or hereditary complex motor and sensory axonal neuropathy (HMSN) with microcephaly.^7^, ^8^, ^9^ However, the appearance of pontocerebellar hypoplasia or microcephaly has not been a consistent phenotype associated with *VRK1* variants. Juvenile‐onset distal hereditary motor neuropathy (dHMN),^10^ adult‐onset distal SMA,^11^, ^12^ and adult‐onset motor neuron disease 13 without pontocerebellar hypoplasia or microcephaly have been associated with *VRK1* variants.

Here we report a homozygous splice variant in *VRK1* in a family, including five affected individuals with childhood‐onset SMA or distal motor neuropathy with brisk tendon reflexes without pontocerebellar hypoplasia and with normal intellectual ability. The variant causes decreased splicing efficiency and a mRNA frameshift that leads to a premature termination codon.

## Materials and Methods

### Ethical approval

The study was approved by the ethical standards of the relevant institutional review board, the Ethics Review Committee in the Gothenburg Region (Dn1: 842‐14) and the Human Research Ethics Committee of the University of Western Australia. Informed consent was obtained from all patients, parents, and family members included in this study after appropriate genetic counseling. Blood samples were obtained from patients and their family members.

### Clinical evaluation

**Figure 1:**
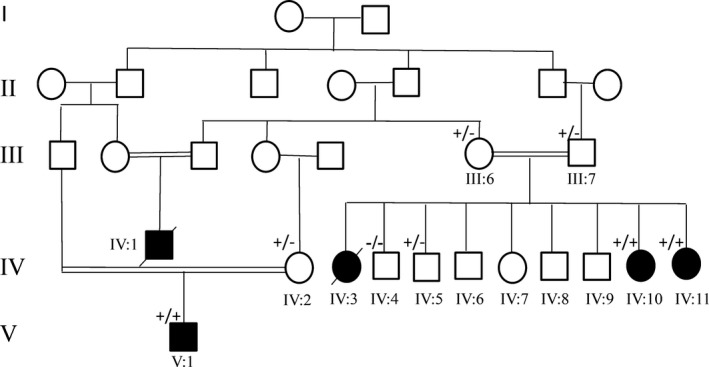
Pedigree of the family and clinical features. Pedigree and recessive inheritance of *VRK1*. In the pedigree, squares represent males; circles, females; open symbols, unaffected family members; and slash, deceased. The affected individuals are represented with shaded symbol. −/− indicates WT, −/+ indicates heterozygous presence of the variant and +/+ indicates homozygous appearance of the variant.

Medical history, physical examination, and imaging were performed as part of routine clinical workup. For the three *VRK1* mutation‐positive patients available for study (Fig. 1), extensive clinical follow‐up was performed. The clinical evaluation was performed by experienced neurologists. Cranial MRI scans were performed in all affected individuals and were evaluated by a neuroradiologist. Neurophysiological studies including sensory and motor neurography and concentric needle electromyography were carried out in the affected individuals.

### Genetic analysis

Prior to this study, the affected individuals (IV:10, IV:11, and V:1) were prescreened for deletion of exon 7 in *SMN1*. Whole‐exome sequencing was performed on DNA from two affected (IV:10 and V:1) and one unaffected (IV:5) individuals in the family, as previously described.^14^ Bidirectional Sanger sequencing was performed in the patients and their unaffected parents and siblings.

### Transcript analysis

To analyze the impact of the c.1159 + 1G>A variant in splicing efficiency of *VRK1* exon 12 in the affected individuals (IV:10, IV:11, and V:1), PCR was performed on cDNA extracted from isolated mRNA from blood with primer pairs covering part of exon 9 through exon 13, spanning the exon 12‐13 junction, and 3’ untranslated region (UTR) (701bp).

### Data availability

The data supporting the findings of this study are available within the article or from the corresponding author on request.

## Results

### Clinical characteristics of patients

We report three sisters (Cases IV:3, IV:10, and IV:11), a cousin (Case IV:1) and a 13‐year‐old second cousin (Case V:1) from a Caucasian family with consanguinity (Fig. 1). They presented with progressive muscle weakness and atrophy. Family history was positive with neurological disorders and occurrence of death in two cases (IV:1 and IV:3).

Case IV:3, a female, was the first child of an apparently healthy first cousin couple (Fig. 1). She presented with similar clinical features as her younger sisters (Cases IV:10 and IV:11), described below, and she died at the age of 32 because of respiratory difficulty.

Case IV:10 is a 33‐year‐old female, the second affected child of this family (Fig. 1). Pregnancy and delivery were unremarkable. She achieved normal motor milestones in childhood. At 12 years of age, she presented with progressive distal muscle weakness of the lower limb that progressed to severely involve proximal and distal muscles of four limbs and she became wheelchair bound at age 25 years. She had completely normal mental activity. Neurological examination revealed global areflexia. The sensory exam was normal. Eye movements were normal. She had scoliosis, bilateral equinovarus, and distal joint hyperlaxity. She had widespread fasciculations. There was no bulbar involvement.

**Figure 2:**
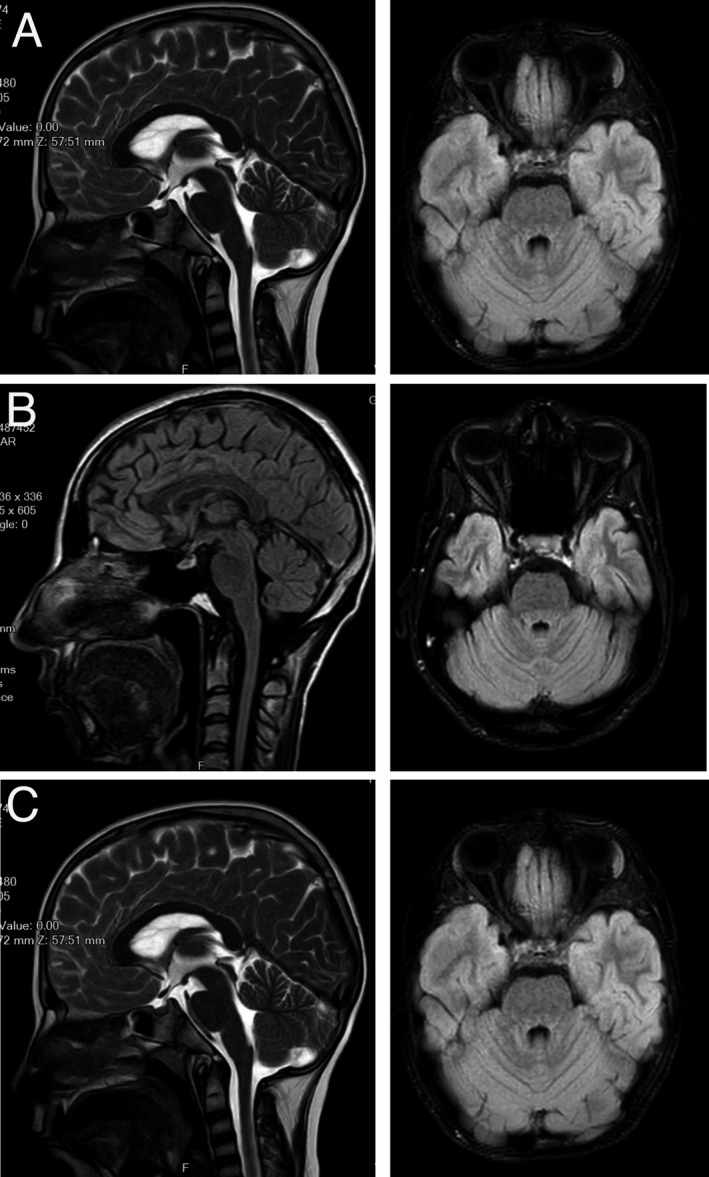
Sagittal T1‐weighted MRI of brain. Axial and sagittal Fluid‐attenuated inversion recovery (FLAIR) brain MRI images of Cases (A) IV:10, (B) IV:11, and (C) V:1 reveal normal cerebellum and indicate no abnormality.

Cardiac examination was normal. At 33 years of age, brain magnetic resonance imaging (MRI) was normal (Fig. 2A).

Sensory nerve conduction parameters were normal. Absent compound muscle action potential (CMAP) in the lower limbs and very low amplitude in upper limbs were observed. The electromyography (EMG) evaluation indicated chronic neurogenic changes with high‐amplitude motor unit action potentials (MUAP) in all tested muscles. Together, results of neurophysiological examination were consistent with predominantly distal SMA.

Case IV:11, a female aged 29 years, is the third affected offspring of this family. She was born after an uncomplicated full‐term pregnancy and delivery. She acquired normal motor milestones. At 6 years of age, she started to develop a progressive muscle weakness of the lower limbs that eventually progressed to involve the four limbs and she became wheelchair bound at the age of 18 years old. She had respiratory distress and respiratory insufficiency from 24 years of age. Similar to her older sister (Case IV:10), she showed normal intellectual development. Brain MRI was normal at age 29 years (Fig. 2B). Neurological examination revealed absent DTRs in all extremities. Sensory exam was normal and the Babinski sign was absent. She had normal eye movements. She had scoliosis, severe lordosis, equinovarus, and distal joint hyperlaxity. She had urinary incontinence from the age of 20 years. Nerve conduction velocity (NCV) and EMG, revealed absence of response in peroneal and tibial nerves and left ulnar nerve and a decreased amplitude in both median nerves and right ulnar nerve. The sensory nerve conduction study was normal. The EMG revealed chronic denervation in all tested muscles. The clinical and electrodiagnostic findings suggested distal SMA.

The affected sisters had six other siblings with normal motor and intellectual development by history.

Case IV:1 was a male and the only child of an apparently healthy first cousin couple of this family (Fig. 1). He died at the age of 17 because of respiratory difficulty.

Case V:1 is a 13‐year‐old male, the only child of a healthy second cousin couple of this family. Unlike his cousins, individuals IV:10 and IV:11, his motor developmental was delayed. He sat at 12 months of age and was assisted walking at 14 months of age. The first clinical examination at 6 years of age, revealed distal muscle weakness of the lower limbs, that later, spread to proximal and distal muscles of four limbs. His mother described episodic fever, mostly at nights, followed by worsening of muscle functions. He became wheelchair bound at the age of 13. He attended regular school and showed normal intellectual development. Brain MRI was normal at age 13 years (Fig. 2C). Neurological examination at 13 years of age revealed hyperactive deep tendon reflexes (DTRs). Sensory exam was normal. He had normal eye movements. Similar to his cousins (IV:10 and IV:11), he had skeletal deformity including scoliosis, lordosis, and equinovarus. The DTRs were brisk in all extremities. The cranial nerves were intact. Fasciculations were not observed. The motor conduction study revealed absence of response in right ulnar, both peroneal nerves and left tibial nerve. The sensory nerve conduction studies were normal. The EMG showed chronic denervation in all tested muscles including left quadriceps, left tibialis anterior, and left first dorsal interosseous. He was diagnosed with motor neuron disease with brisk tendon reflexes mimicking juvenile ALS.

### Genetic findings

**Figure 3:**
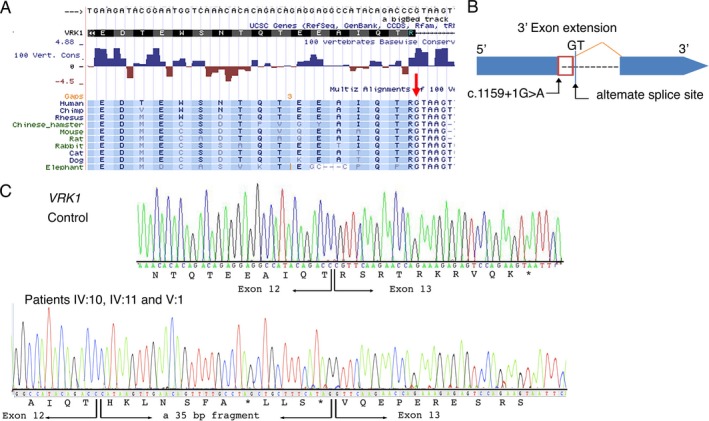
Genetic findings. (A) Species conservation of amino acids and the highly conserved GT nucleotides (red arrow) of the 5’ splice junction of intron 12 of *VRK1* (shaded). (B) *In silico* prediction analysis (MaxEntScan) indicated the likely use of the nearby alternative splice site located 35 bases (red box) from the exon/intron boundary on the 3’ side upstream of exon 13. (C) Sanger sequencing of reverse transcriptase PCR on whole‐blood tissue of the patients (IV:10, IV:11, and V:1) revealed the presence of *VRK1* messenger RNA variant in the patients. A larger fragment in the patients indicated a amplicon covering exon 12 and the entire exon 13 with the insertion of a 35 bp fragment from the exon/intron boundary on the 3’ side upstream of exon 13 and a mRNA frameshift and subsequently to a premature termination codon.

Data from whole‐exome sequencing on DNA from individuals IV:10, IV:5, and V:1 were analyzed through the use of the Ingenuity Variant Analysis (IVA) software (Qiagen, Hilden Germany). The filtering strategy initially concentrated on homozygous coding variants in known neurogenetic disease genes. A novel homozygous G to A change affecting a highly conserved nucleotide of the 5’ splice junction of intron 12 (c.1159 + 1G>A) of *VRK1* (NM_003384.2) was identified in individuals IV:10 and V:1 (Fig. S1A and Fig. 3A). The variant was identified in heterozygous state in individual IV:5.

The *VRK1* variant was validated by PCR and bidirectional Sanger sequencing analysis in individuals IV:10, IV:5, and V:1 (Fig. S1B). The variant is not present on any allele in the Genome Aggregation Database (gnomAD, ExAC or 1000 Genome Project Database (1000G)). *In silico* combined annotation‐dependent depletion (CADD) analysis of the variant revealed high‐deleterious scores: 25.200. *In silico* analysis predicted the *VRK1* variant to be disease causing, presumably identified as potentially affecting splicing (MutationTaster, http://mutationtaster.org/). In addition, *in silico* prediction with the Human Splicing Finder version 3.0 and MaxEntScan suggested that the G to A nucleotide changes the highly conserved GU end in the 5’ splice donor site of intron 12 of *VRK1* and would result in a decreased RNA splicing efficiency of exon 12 by 90% (from 9.09 to 0.91). *In silico* transcript prediction analysis (MaxEntScan) further indicated that the variant would result in the use of a nearby cryptic alternative splice site located 35 bases from the exon/intron boundary on the 3’ side. This would cause a mRNA frameshift and subsequently lead to a premature termination codon (Fig. 3B).

The appearance of the *VRK1* variant was examined in all available family members by Sanger sequencing analysis, which confirmed segregation of the homozygous variant with the disease phenotype. The asymptomatic parents and siblings were either heterozygous or wild‐type for the *VRK1* variant (Fig. S1B).

### Transcript analysis

Owing to the location of the variant, at the last exon‐exon junction, the nonsense‐mediated decay (NMD) machinery is not expected to be engaged to initiate degradation of the mutant messenger RNA, and yield a stable mRNA that directs the synthesis of C‐terminally truncated polypeptides.^15^, ^16^ To determine the actual effect of the homozygous c.1159 + 1G>A variant on *VRK1* gene expression and transcript levels, analysis of the *VRK1* mRNA was performed by PCR on complementary DNA (cDNA) extracted from blood samples from the available individuals in the family. The RT‐PCR analysis indicated detectable expression levels of *VRK1* transcript in the affected individuals IV:10, IV:11, and V:1. While amplification of the cDNA fragment, covering exon 9 through to the 3’UTR, resulted in a single amplicon of 701bp in controls, a larger fragment (736 bp) was detectable in the three affected individuals. Sanger sequencing of the cDNA fragment in the patients indicated a full‐length amplicon covering exon 12 and the entire exon 13, but with the insertion of a 35 bp fragment from the exon/intron boundary on the 3’ side upstream of exon 13 (Fig. 3C). This indicates an inefficient RNA splicing of exon 12 leading to a frameshift, but synthesis of a stable mRNA which escapes the NMD machinery. Subsequently, this would lead to a premature termination codon, p.Arg387Hisfs*7 (Fig. 3C).

We were, however, not able to determine whether the *VRK1* transcript results in production of a stable truncated VRK1 protein or an unstable protein, due to lack of a specific VRK1 antibody suitable for immune blot analysis.

## Discussion

Due to advances in genomic testing, the list of underlying genetic causes of many forms of hereditary motor neuron disease will continue to grow.^17^ Here, we report a homozygous splice variant in *VRK1* and the presence of neurological conditions in a family, including five individuals and occurrence of death in two cases. The affected individuals presented with progressive muscle weakness and atrophy and clinical examination suggested childhood‐onset SMA or juvenile motor neuron disease with hyperreflexia without pontocerebellar hypoplasia or microcephaly and normal intellectual ability.

The ubiquitously expressed VRK1 is a serine/threonine protein kinase that phosphorylates p53 and transcription factor cAMP response element‐binding (CREB) protein. It is essential for cell cycle regulation, histone modification, chromatin organization, and nuclear envelope formation.^18^, ^19^, ^20^ In addition, it has a vital role in embryonic cortical neuronal proliferation and migration through amyloid‐ß precursor protein (APP) and has increased expression in proliferating cells.^6^

**Figure 4:**
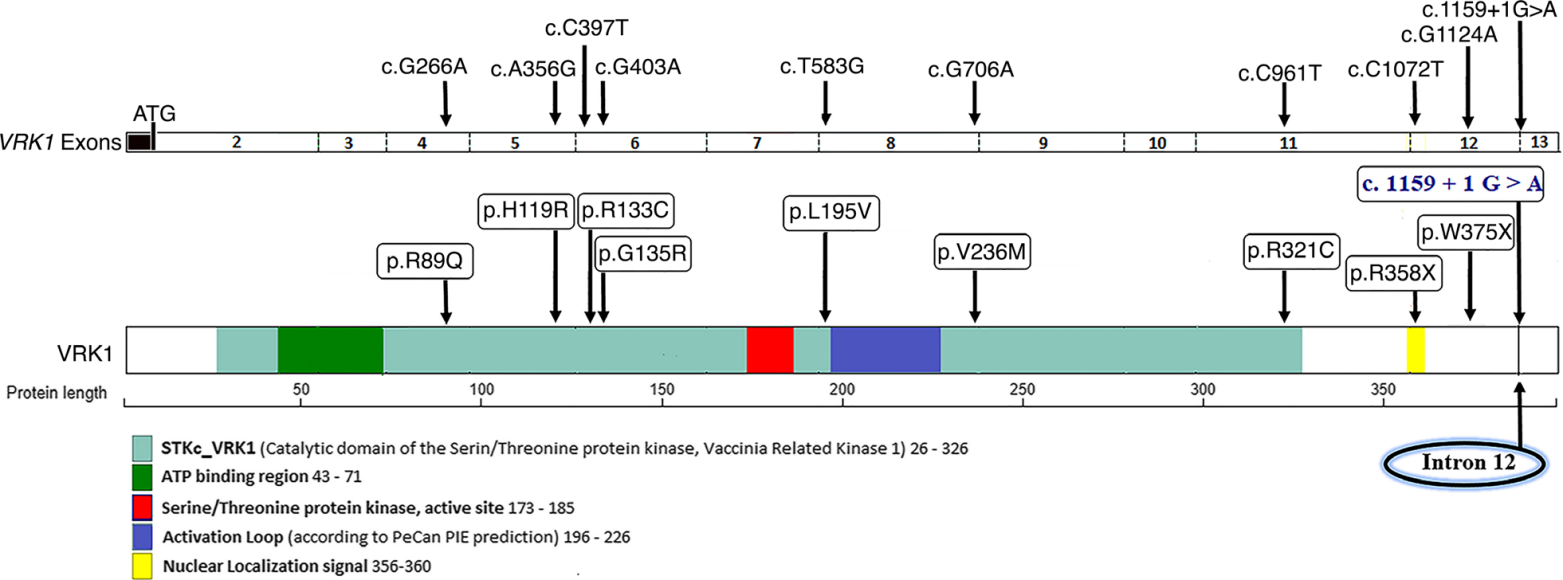
Distribution of *VRK1* variants in previously reported patients and currently described recessive variant. Schematic representation of the *VRK1* transcript and VRK1 protein, including the catalytic domain of the ATP‐binding site, active site of Serine/Threonine protein kinase, activation loop, and nuclear localization signal. Previously reported homozygous (p.R133C, p.R358X, and p.W375X) and compound heterozygous (p.R89Q and p.V236M; p.H119R and p.R321C; p.H119R and p.R358X; and p.G135R and p.L195V) variants (black color) and the currently homozygous c.1159 + 1G>A (blue color) situated at the highly conserved 5’ splice junction of intron 12 are indicated. To date the genetic null variants are C‐terminal, the missense variants more N‐terminal.

Variants in *VRK1* have been segregated with motor neuron diseases including SMA phenotype, adult‐onset motor neuron disease, HMSN, and juvenile‐onset dHMN with or without pontocerebellar hypoplasia or microcephaly 7, 8, 9, 10, 11, 12, 13 (Table S1 and Fig. 4). A homozygous nonsense mutation in *VRK1* (p.R358X) has been described to cause an infantile onset SMA‐PCH phenotype associated with progressive microcephaly of prenatal onset, mental deficiency, significant ataxia, and death in infancy or childhood in a consanguineous family of Ashkenazi Jewish origin.^7^ The same recessive nonsense mutation was reported in a patient of Ashkenazi Jewish origin with a HMSN accompanied by severe nonprogressive microcephaly and cerebral dysgenesis of prenatal onset.^8^ In addition, homozygous missense mutation in *VRK1* (p.R133C) has been associated with SMA‐PCH in a family with four affected siblings 9 and compound heterozygous missense mutations (p.R89Q and p.V236M) have been identified in two siblings with HMSN and severe nonprogressive microcephaly.^8^ Furthermore, compound heterozygous missense mutations (p.G135R and p.L195V) have been identified in a patient with juvenile motor neuron disease and microcephaly.^11^ Segregation of *VRK1* variants in patients with SMA phenotype or motor neuron disease without pontocerebellar hypoplasia or microcephaly has also been reported. Compound heterozygosity for the same nonsense mutation and a novel missense mutation (p.R358X and p.H119R) in two siblings of Ashkenazi Jewish ancestry and homozygosity for a novel nonsense mutation (p.W375X) in two Chinese siblings with adult‐onset distal SMA and a Chinese patient with Juvenile‐onset dHMN without pontocerebellar hypoplasia have been reported.^10^, ^11^, ^12^ Furthermore, compound heterozygous missense mutations (p.H119R and p.R321C) have been identified in a patient with adult‐onset motor neuron disease without pontocerebellar hypoplasia or microcephaly 13 (Fig. 4 and Table S1).

Development of pontocerebellar hypoplasia or microcephaly in motor neuron disease patients associated with *VRK1* has not been a frequent finding in reported cases, with seven patients so far,^7^, ^8^, ^9^, ^11^ and it is not a clinical feature in the family reported here. It thus remains unknown whether patients with VRK1‐associated motor neuron disease have a predisposition to pontocerebellar hypoplasia or microcephaly, given the few patients with *VRK1* mutation that have been described so far and the lack of functional study associated with the reported variants. There is further no clear correlation between the location of the *VRK1* variant and the associated clinical features (Fig. 4, Tables [Link], [Link] and [Link], [Link]). In order to understand the pathological basis of the VRK1‐associated diseases, a greater understanding of the roles of the VRK1 and its subdomains in normal function is needed.

Consistent with the *in silico* analysis prediction, results from our transcript assay in the patients revealed the production of a larger *VRK1* transcript with insertion of a 35 bp fragment from the exon/intron boundary that escapes the NMD machinery, leading to a mRNA frameshift and subsequently to a premature termination codon. To the best of our knowledge, this is the first study enlightening the impact of a *VRK1* variant on the transcript production in the patients. Nevertheless, the downstream consequences of the *VRK1* variant on the VRK1 production remain unknown. Further studies are required to investigate the regulation of *VRK1* and the role of the VRK1 protein in neuronal function to understand which aspect of the VRK1 protein function are affected to lead to motor neuron diseases with or without pontocerebellar hypoplasia or microcephaly and intellectual disability/central nervous system involvement.

## Web Resources

The following databases were used in this study: The Exome Variant Server: NHLBI Exome Sequencing Project (ESP), Seattle, WA; URL: http://evs.gs.washington.edu/EVS/. 1000 Genome Project Database: http://browser.1000genomes.org/index.html. Human Background Variant DataBase: http://neotek.scilifelab.se/hbvdb/. Genome Aggregation Database (GnomAD): http://gnomad.broadinstitute.org/. Greater Middle East (GME) Variome web: http://igm.ucsd.edu/gme/index.php. Ensembl genome browser: http://www.ensembl.org/. MutationTaster: http://mutationtaster.org/. MaxEntScan**:** http://genes.mit.edu/burgelab/maxent/Xmaxentscan_scoreseq.html.

## Consent to Publish

The parents in this study provided written informed consent to publish their family trees, and family data, including photographs.

## Conflict of Interests

The authors declare that they have no competing interests.

## Supporting information


**Figure S1.** Whole exome and Sanger sequencing. (A) The plus strand of the genome around the *VRK1* variant (red arrow) demonstrates the presence of the c.1159 + 1G>A variant in Case IV:10. (B) Sanger sequence analysis of the genomic DNA demonstrates the segregation of the *VRK1* c.1159 + 1G>A variant (red arrow) in the family.

**Table S1.** Clinical features of the patients with *VRK1* variants.

**Table S2.** VRK1 genotype/phenotype correlation.

## References

[1] Lefebvre S , Burglen L , Reboullet S , et al. Identification and characterization of a spinal muscular atrophy‐determining gene. Cell 1995;80:155–165. 7813012 10.1016/0092-8674(95)90460-3

[2] Moultrie RR , Kish‐Doto J , Peay H , Lewis MA . A review on spinal muscular atrophy: awareness, knowledge, and attitudes. J Genet Couns 2016;25:892–900. 27084745 10.1007/s10897-016-9955-8

[3] Zerres K , Rudnik‐Schoneborn S . 93rd ENMC international workshop: non‐5q‐spinal muscular atrophies (SMA) ‐ clinical picture (6–8 April 2001, Naarden, The Netherlands). Neuromuscul Disord 2003;13:179–183. 12565918 10.1016/s0960-8966(02)00211-0

[4] Barth PG . Pontocerebellar hypoplasias. An overview of a group of inherited neurodegenerative disorders with fetal onset. Brain Dev 1993;15:411–422. 8147499 10.1016/0387-7604(93)90080-r

[5] Rudnik‐Schoneborn S , Sztriha L , Aithala GR , et al. Extended phenotype of pontocerebellar hypoplasia with infantile spinal muscular atrophy. Am J Med Genet A 2003;117A:10–17. 12548734 10.1002/ajmg.a.10863

[6] Vinograd‐Byk H , Sapir T , Cantarero L , et al. The spinal muscular atrophy with pontocerebellar hypoplasia gene VRK1 regulates neuronal migration through an amyloid‐beta precursor protein‐dependent mechanism. J Neurosci 2015;35:936–942. 25609612 10.1523/JNEUROSCI.1998-14.2015 PMC6605533

[7] Renbaum P , Kellerman E , Jaron R , et al. Spinal muscular atrophy with pontocerebellar hypoplasia is caused by a mutation in the VRK1 gene. Am J Hum Genet 2009;85:281–289. 19646678 10.1016/j.ajhg.2009.07.006 PMC2725266

[8] Gonzaga‐Jauregui C , Lotze T , Jamal L , et al. Mutations in VRK1 associated with complex motor and sensory axonal neuropathy plus microcephaly. JAMA Neurol 2013;70:1491–1498. 24126608 10.1001/jamaneurol.2013.4598 PMC4039291

[9] Najmabadi H , Hu H , Garshasbi M , et al. Deep sequencing reveals 50 novel genes for recessive cognitive disorders. Nature 2011;478:57–63. 21937992 10.1038/nature10423

[10] Feng SY , Li LY , Feng SM , Zou ZY . A novel VRK1 mutation associated with recessive distal hereditary motor neuropathy. Ann Clin Transl Neurol 2019;6:401–405. 30847374 10.1002/acn3.701 PMC6389749

[11] Stoll M , Teoh H , Lee J , et al. Novel motor phenotypes in patients with VRK1 mutations without pontocerebellar hypoplasia. Neurology 2016;87:65–70. 27281532 10.1212/WNL.0000000000002813 PMC4932233

[12] Li N , Wang L , Sun X , et al. A novel mutation in VRK1 associated with distal spinal muscular atrophy. J Hum Genet 2019;64:215–219. 30617279 10.1038/s10038-018-0553-5

[13] Nguyen TP , Biliciler S , Wiszniewski W , Sheikh K . Expanding Phenotype of VRK1 Mutations in Motor Neuron Disease. J Clin Neuromuscul Dis 2015;17:69–71. 26583493 10.1097/CND.0000000000000096 PMC4829393

[14] Kariminejad A , Dahl‐Halvarsson M , Ravenscroft G , et al. TOR1A variants cause a severe arthrogryposis with developmental delay, strabismus and tremor. Brain 2017;140:2851–2859. 29053766 10.1093/brain/awx230

[15] Nagy E , Maquat LE . A rule for termination‐codon position within intron‐containing genes: when nonsense affects RNA abundance. Trends Biochem Sci 1998;23:198–199. 9644970 10.1016/s0968-0004(98)01208-0

[16] Thermann R , Neu‐Yilik G , Deters A , et al. Binary specification of nonsense codons by splicing and cytoplasmic translation. EMBO J 1998;17:3484–3494. 9628884 10.1093/emboj/17.12.3484 PMC1170685

[17] Wee CD , Kong L , Sumner CJ . The genetics of spinal muscular atrophies. Curr Opin Neurol 2010;23:450–458. 20733483 10.1097/WCO.0b013e32833e1765

[18] Vega FM , Sevilla A , Lazo PA . p53 Stabilization and accumulation induced by human vaccinia‐related kinase 1. Mol Cell Biol 2004;24:10366–10380. 15542844 10.1128/MCB.24.23.10366-10380.2004 PMC529057

[19] Valour D , Ochala J , Ballay Y , Pousson M . The influence of ageing on the force‐velocity‐power characteristics of human elbow flexor muscles. Exp Gerontol 2003;38:387–395. 12670625 10.1016/s0531-5565(02)00265-6

[20] Salzano M , Sanz‐Garcia M , Monsalve DM , et al. VRK1 chromatin kinase phosphorylates H2AX and is required for foci formation induced by DNA damage. Epigenetics 2015;10:373–383. 25923214 10.1080/15592294.2015.1028708 PMC4623420

