# Supplementary figures and images for: Motor neuron diseases caused by a novel VRK1 variant – A genotype/phenotype study
Source: Ann Clin Transl Neurol. 2019 Sep 27;6(11):2197–204. doi: 10.1002/acn3.50912 (PMC6856620; doi:10.1002/acn3.50912)

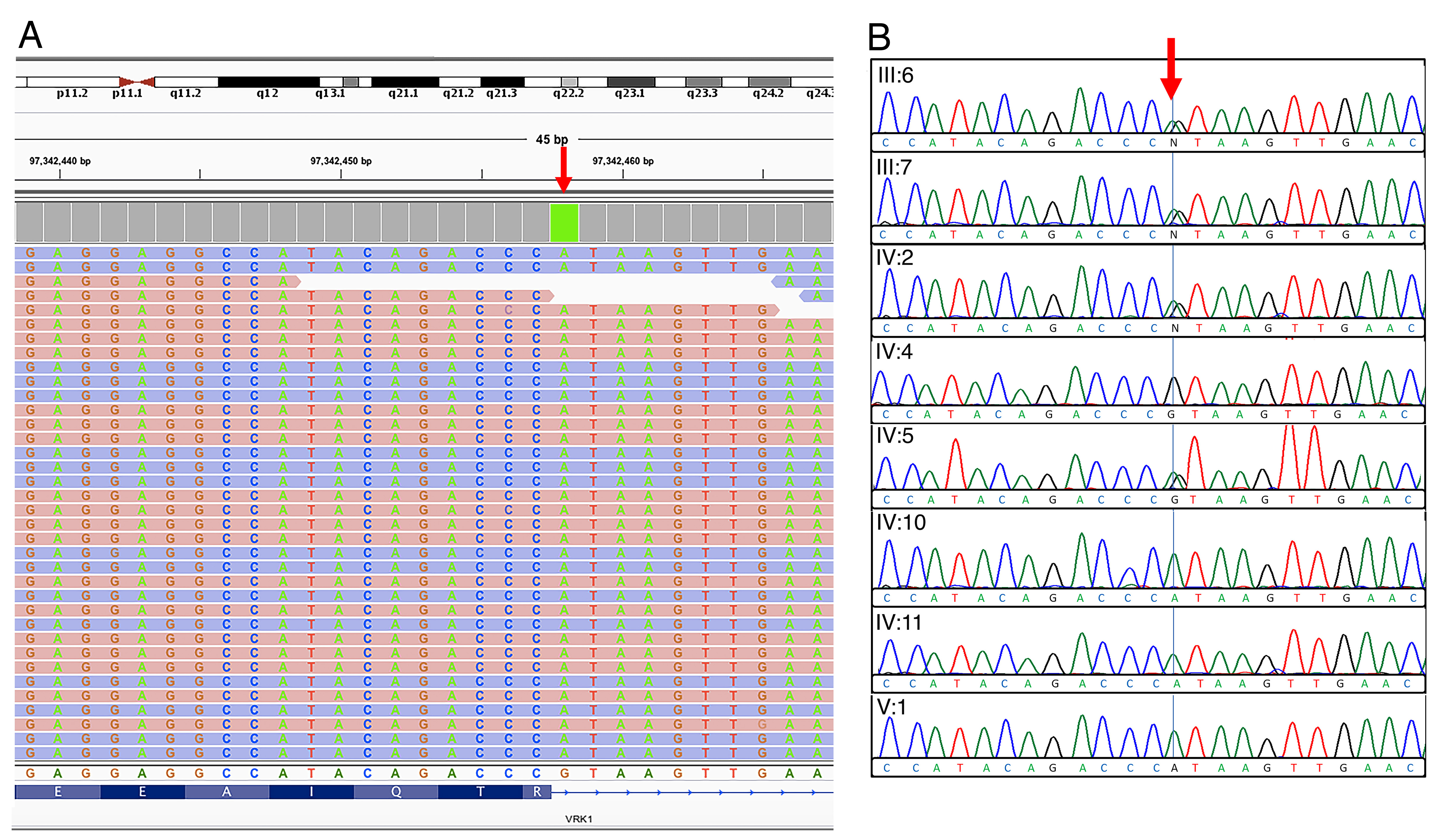

Supplement: Supplementary file 1 — Figure S1. Whole exome and Sanger sequencing. (A) The plus strand of the genome around the VRK1 variant (red arrow) demonstrates the presence of the c.1159 + 1G>A variant in Case IV:10. (B) Sanger sequence analysis of the genomic DNA demonstrates the segregation of the VRK1 c.1159 + 1G>A variant (red arrow) in the family. [file ACN3-6-2197-s001.tif]
