# Supplementary material for: Motor neuron diseases caused by a novel VRK1 variant – A genotype/phenotype study
Source: Ann Clin Transl Neurol. 2019 Sep 27;6(11):2197–204. doi: 10.1002/acn3.50912 (PMC6856620; doi:10.1002/acn3.50912)
Supplement: Supplementary file 2 — Table S1. Clinical features of the patients with VRK1 variants. [file ACN3-6-2197-s002.docx]

**Supplemental Table 1:** Clinical features of the patients with *VRK1* variants

| **Study** | **Renbaum et al. (2009)** | | **Najmabadiet al, (2011)** | **Gonzaga-Jauregui et al.**  **(2013)** | | | **Nguyen et al       (2015)** | **Stoll et al.**  **(2016)** | | | **Li et al.**  **(2018)** | | **Feng et al. (2019)** | **Present study** | | |
| --- | --- | --- | --- | --- | --- | --- | --- | --- | --- | --- | --- | --- | --- | --- | --- | --- |
| Phenotype | SMA-PCH | | SMA-PCH | HMSN & microcephaly | | HMSN & prenatal-onset microcephaly | Sporadic Adult-onset motor neuron disease | Adult-onset distal SMA | | Juvenile motor neuron disease and microcephaly | Adult-onset distal SMA | | Juvenile-onset hereditary motor neuropathy | Adult-onset  distal SMA | | Lower motor neuron disease with brisk reflexes |
| Origin | Ashkenazi, Jewish | | Iranian | NM | | Ashkenazi, Jewish | NM | Ashkenazi, Jewish | | NM | Chines | | Chines | Iranian | | Iranian |
| *VRK1* variants  (NM_003384.2) | Homozygous c.C1072T (p.R358X) | | Homozygous c.C397T (p.R133C) | Compound heterozygous  c.G266A (p.R89Q) & c.G706A (p.V236M) | | Homozygous c.C1072T (p.R358X) | Compound heterozygous c.A356G (p.H119R) &  c.C961T (p.R321C) | Compound heterozygous  c.A356G (p.H119R)  & c.C1072T (p.R358X) | | Compound heterozygous c.G403A (p.G135R) & c.T583G (p.L195V) | c.1124G >A, p.W375X | | c.1124G >A, p.W375X | Homozygous  c.1159+1G>A | | Homozygous  c.1159+1G>A |
| Affected domain | KKRKK C-terminal NLS | | Kinase domain | Kinase domain | | KKRKK C-terminal NLS | Kinase domain | KKRKK C-terminal NLS  & Kinase domain | | Kinase domain | BAD motif near to NLS at the C terminus | | BAD motif near to NLS at the C terminus | C-terminus | | C-terminus |
| Individual ID | IV-12 | IV-6 | M017N  (4 siblings) | BAB3022 | BAB3280 | BAB5311 | _ | II:4 | II:1 | III:4 | II:7 | II:9 | V:1 | IV:10 | IV:11 | V:1 |
| Microcephaly | + | + | NM | + | + | + | **-** | **-** | **-** | + | NM | NM | - | - | - | - |
| Intellectual disability | + | + | + | - | - | - | - | - | - | NM | - | - | - | - | - | - |
| Motor milestone development | Delayed | Delayed | NM | Delayed | Delayed | Delayed | Normal | Normal | Normal | Delayed | Normal | Normal | NM | Normal | Normal | Delayed |
| Progressive muscle wasting | + | + | NM | + | NM | + | + | + | + | + | + | + | + | + | + | + |
| Respiratory insufficiency | _ | _ | NM | + | NM | NM | _ | + | + | + | NM | NM | NM | + | + | _ |
| Ataxia | + | + | NM | _ | _ | _ | NM | _ | _ | NM | _ | _ |  | _ | _ | _ |
| Deep tendon reflex (DTR) | Brisk | Brisk | NM | Absent | Normal | Decreased | Brisk | Lower limbs: Absent  Upper limbs: Brisk | | Brisk | Absent | Lower limbs: absent  Upper limbs: Brisk | Lower limbs: absent  Upper limbs: normal | Absent | Absent | Brisk |
| Electrophysiological studies | Motor and sensory neuropathy.Chronic and active denervation/reinnervation | Demyelinating peripheral neuropathy with chronic denervation | NM | Axonal motor and sensory peripheral neuropathy | Axonal motor and sensory neuropathy | | Normal sensory neurography. Chronic and active denervation/reinnervation | Normal sensory neurography. Chronic and active denervation/reinnervation | | Normal sensory neurography, second examination:Reducedsensory action potentials.  Chronic and active denervation/reinnervation | Normal sensory neurography,chronic denervation/reinnervation | | Pure motor axonalneuropathy of the lower limbs with preserved conduction velocities and no sensory involvement | Normal sensory neurography.  Widespread chronic denervation/reinnervation | | Normal sensory neurography. Chronic and active  denervation/reinnervation |
| Scoliosis | NM | NM | NM | + | _- | + | NM | _- | _- | + | NM | | NM | + | + | + |
| Brain and spine MRI/  Brain CT | Small vermis;  Large cisterna magna compatible with cerebellar hypoplasia | cerebellar hypoplasia with suspected pontine  hypoplasia | NM | Microcephaly and a simplified gyral pattern | Microcephaly and a simplified gyral pattern | Microcephaly and a simplified gyralpattern and  underdeveloped cerebellar vermis | Normal | Spinal cord atrophy, non-specific mild to moderate generalized brain atrophy | | Normal | Normal | Normal except for surgical defect | Normal | Normal | Normal | Normal |
| Others | Swallowing difficulties. | Swallowing difficulties  and bladder disturbances |  |  |  |  | Elevated serum CK |  |  | Short stature | Family cancer history | Right cerebellar hemangioblastoma,  mildly elevated CK & family cancer history | Right and left foot drop | Episodic fever and headache was concurrent with the onset of disease | | Episodic fever mostly at nights |

NM: Not Mentioned
