# Supplementary material for: Motor neuron diseases caused by a novel VRK1 variant – A genotype/phenotype study
Source: Ann Clin Transl Neurol. 2019 Sep 27;6(11):2197–204. doi: 10.1002/acn3.50912 (PMC6856620; doi:10.1002/acn3.50912)
Supplement: Supplementary file 3 — Table S2. VRK1 genotype/phenotype correlation. [file ACN3-6-2197-s003.docx]

**Supplemental Table 2**: VRK1 genotype/phenotype correlation

| **Variants** | **Zygosity** | **Adult onset motor neuron disease** | **Adult onset distal SMA** | **HMSN** | **Juvenile-onset dHMN** | **Lower motor neuron disease with brisk reflexes** | **Microcephaly** | **SMA-PCH** |
| --- | --- | --- | --- | --- | --- | --- | --- | --- |
| R89Q/V236M | Compound hetero |  |  | √ |  |  | √ |  |
| H119R/R321C | Compound hetero | √ |  |  |  |  |  |  |
| H119R/R358X | Compound hetero |  | √ |  |  |  |  |  |
| G135R/L195V | Compound hetero |  |  |  |  | √ | √ |  |
| R113C | Homo |  |  |  |  |  |  | √ |
| R358X | Homo |  |  |  |  |  |  | √ |
| R358X | Homo |  |  | √ |  |  | √ |  |
| W375X | Homo |  | √ |  | √ |  |  |  |
| c.1159+1G>A | Homo |  | √ |  |  |  |  |  |
| c.1159+1G>A | Homo |  |  |  |  | √ |  |  |

There is no obvious genotype/phenotype correlation
